# Antibody Persistence 1–5 Years Following Vaccination With MenAfriVac in African Children Vaccinated at 12–23 Months of Age

**DOI:** 10.1093/cid/civ672

**Published:** 2015-11-09

**Authors:** Milagritos D. Tapia, Helen Findlow, Olubukola T. Idoko, Marie-Pierre Preziosi, Prasad S. Kulkarni, Godwin C. Enwere, Cheryl Elie, Varsha Parulekar, Samba O. Sow, Fadima Cheick Haidara, Fatoumata Diallo, Moussa Doumbia, Adebayo K. Akinsola, Richard A. Adegbola, Beate Kampmann, Julie Chaumont, Lionel Martellet, Elisa Marchetti, Simonetta Viviani, Yuxiao Tang, Brian D. Plikaytis, F. Marc LaForce, George Carlone, Ray Borrow

**Affiliations:** 1 Department of Pediatrics, Center for Vaccine Development, University of Maryland School of Medicine, Baltimore; 2 Vaccine Evaluation Unit, Public Health England, Manchester Royal Infirmary, United Kingdom; 3 Vaccines and Immunity Theme, Medical Research Council Unit, Basse, The Gambia; 4 Meningitis Vaccine Project, PATH, Ferney-Voltaire, France; 5 Meningitis Vaccine Project, Department of Immunization, Vaccines and Biologicals, World Health Organization, Geneva, Switzerland; 6 Serum Institute of India, Ltd, Pune; 7 Centers for Disease Control and Prevention, Atlanta, Georgia; 8 DiagnoSearch Life Sciences, Mumbai, India; 9 Centre pour le Développement des Vaccins, Ministère de la Santé, Bamako, Mali; 10 GlaxoSmithKline Vaccines, Wavre, Belgium; 11 Meningitis Vaccine Project, PATH, Seattle, WA

**Keywords:** MenAfriVac, group A meningococcal conjugate vaccine, antibody persistence, African meningitis belt

## Abstract

***Background.*** Following mass vaccination campaigns in the African meningitis belt with group A meningococcal conjugate vaccine, MenAfriVac (PsA-TT), disease due to group A meningococci has nearly disappeared. Antibody persistence in healthy African toddlers was investigated.

***Methods.*** African children vaccinated at 12–23 months of age with PsA-TT were followed for evaluation of antibody persistence up to 5 years after primary vaccination. Antibody persistence was evaluated by measuring group A serum bactericidal antibody (SBA) with rabbit complement and by a group A–specific IgG enzyme-linked immunosorbent assay (ELISA).

***Results.*** Group A antibodies measured by SBA and ELISA were shown to decline in the year following vaccination and plateaued at levels significantly above baseline for up to 5 years following primary vaccination.

***Conclusions.*** A single dose of PsA-TT induces long-term sustained levels of group A meningococcal antibodies for up to 5 years after vaccination.

***Clinical Trials Registration.*** ISRTCN78147026.

The “meningitis belt” of sub-Saharan Africa runs across the continent from Senegal to Ethiopia, and this region is prone to major epidemics of meningococcal meningitis, with a high case fatality rate and serious sequelae. Until recently, most epidemics were due to group A *Neisseria meningitidis* (MenA). The observed reduction in meningococcal meningitis due to MenA has been due to the development of an affordable group A meningococcal vaccine, PsA-TT (MenAfriVac, Serum Institute of India, Ltd). The Meningitis Vaccine Project, a partnership between the World Health Organization (WHO) and PATH, coordinated the development, testing, licensure, and introduction of this vaccine with the aim of eliminating epidemic meningitis attributable to MenA [1–4].

Since 2010, PsA-TT has been introduced into countries across the meningitis belt through mass campaigns [5, 6]. PsA-TT is expected to confer both longer-lasting individual protection and herd protection than MenA polysaccharide vaccines. Ongoing surveillance shows that no cases of MenA meningococcal disease have occurred in vaccinated individuals [6], but the longevity of this protective immune response is not known. Prior to the introduction of PsA-TT into the African countries of the meningitis belt, multiple clinical trials assessed the safety and immunogenicity of PsA-TT. A trial conducted in children aged 12–23 months demonstrated that this vaccine was immunogenic and induced immune memory. However, antibody persistence is key in maintaining direct and indirect protection [7, 8]. We report here on the persistence of MenA-specific antibodies in individuals vaccinated with PsA-TT in early childhood.

## METHODS

The study was conducted in accordance with the principles of the Declaration of Helsinki and in compliance with Good Clinical Practice guidelines. The clinical trial was registered (identifier ISRCTN78147026) at www.controlled-trials.com.

The full details of this study have been reported elsewhere by Sow et al [9]; in brief, healthy children (aged 12–23 months) who were fully immunized according to the local Expanded Programme on Immunization schedule were recruited from 2 urban quarters in Bamako, Mali, and from Basse in the Upper River region of The Gambia. Subjects received primary vaccination when aged between 12 and 23 months of either PsA-TT (10 µg), polysaccharide vaccine (PsACWY), or *Haemophilus influenzae* type b vaccine (Hib-TT) and 10 months later were revaccinated with 1 of these 3 vaccines (the dose of PsACWY administered was one-fifth dose). Blood samples were obtained prior to primary vaccination and revaccination, 4 weeks after primary vaccination, and 1 and 4 weeks after revaccination, the results of which were previously reported [9]. Those subjects who received Hib-TT at the primary and revaccination stages of the initial trial were vaccinated with PsA-TT at the end of the initial trial (3–4 years of age at approximately 2 years following enrollment). The initial trial period followed subjects for 2 years following primary vaccination. Subjects were later approached for enrollment into a follow-on study to assess the persistence of group A–specific antibodies approximately 5 years after primary vaccination. For evaluation of antibody persistence, blood samples were obtained at approximately 1, 2, and 5 years following primary vaccination (initial trial enrollment) in 3 groups who received (1) a single dose of PsA-TT at either primary or revaccination stages, (2) two doses of PsA-TT, and (3) a single dose of PsA-TT at the end of the initial trial period (at 2 years [104 weeks] after primary vaccination) (those who received 2 doses of Hib-TT).

### Immunogenicity

Blood samples were assayed in the serum bactericidal antibody (SBA) assay using the group A target strain F8238 (phenotype A:4,21:P1.20,9, L10) as previously described [10]. The complement source used in the SBA was pooled serum from 3- to 4-week-old rabbits (Pel Freez Biologicals). Titers were expressed as the reciprocal serum dilutions yielding ≥50% killing after 60 minutes.

Group A–specific immunoglobulin G (IgG) levels were determined using an enzyme-linked immunosorbent assay (ELISA) [11], except that the reference serum CDC1992 and monoclonal-pan antihuman IgG Fc labeled with horseradish peroxidase (Hybridoma Reagent Laboratory) were used. For the reference serum, CDC1992 was used with the previously assigned group A–specific IgG concentration [12]. The lower limit of quantitation for the ELISA was 0.4 µg/mL; concentrations below this were reported as 0.2 µg/mL.

### Statistical Analysis

The SBA geometric mean titers (GMTs) and group A–specific IgG geometric mean concentrations (GMCs) between the vaccine groups at 1 year and 2 years after primary vaccination were compared by mixed-effects modeling adjusted for baseline titers (concentrations), age, sex, study site, time, and interaction effects of interest with log_2_-transformed titers and log_10_-transformed concentrations as an outcome. At 5 years after primary vaccination, the comparisons in SBA GMTs and group A–specific IgG GMCs between the vaccine groups of interest were performed by analysis of covariance adjusted for baseline titers (concentrations), sex, and time. The paired *t* test was used to compare the GMTs and GMCs between 2 and 5 years after primary vaccination. The percentages of subjects with SBA titers ≥128 and group A–specific IgG concentrations ≥2 µg/mL along with their exact binomial 95% confidence intervals (CIs) were provided. All immunogenicity analyses were conducted in the intention-to-treat population. Missing values were treated as missing at random. All tests were 2-sided with a significance level of .05. Data analysis was performed using SAS software, version 9.1.3.

## RESULTS

### Study Population

**Figure 1.**
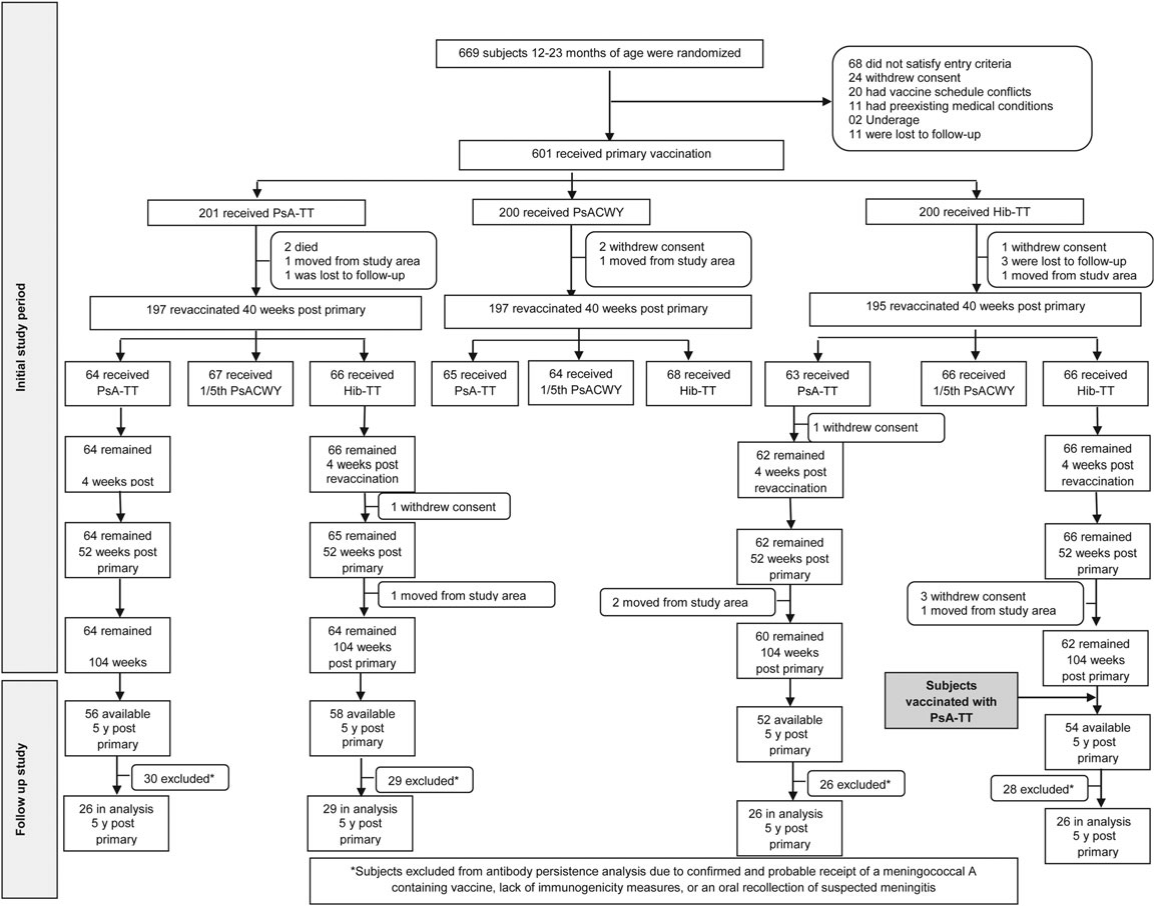
Subject disposition. Abbreviations: Hib-TT, *Haemophilus influenzae* type b–tetanus toxoid vaccine; PsACWY, polysaccharide ACWY vaccine; PsA-TT, meningococcal A polysaccharide–tetanus toxoid protein conjugate vaccine.

As previously reported [9], 601 Malian and Gambian toddlers were randomized to receive primary vaccination, of whom 589 completed the revaccination stage. Subjects who were recruited into the PsA-TT and Hib-TT groups and received either PsA-TT or Hib-TT at the revaccination phase were followed for antibody persistence at approximately 1, 2, and 5 years after primary vaccination. Figure 1 shows the subjects’ disposition for the initial trial period and for the follow-up study period. All 101 subjects from the Mali site were excluded from the 5-year persistence analysis due to the confirmed and probable receipt of MenAfriVac as part of the national mass vaccination campaign in 2010–2011 or trivalent meningococcal ACW vaccine during an outbreak of group W meningococcal disease. Long-term persistence was conducted on average 5 years after the initial trial period with a mean duration of 5.1 years following primary vaccination.

### Serum Bactericidal Antibody Titers

The SBA GMTs and proportion of subjects with SBA titers ≥128 for each study group are shown in Table 1. At 1 year after primary vaccination, the SBA GMTs of the groups that had received PsA-TT were significantly higher than those that had not received any MenA-containing vaccine (Hib-TT/Hib-TT) (*P* < .0001 for all 3 pairwise comparisons).

**Table 1.** Summary of Endpoints of Group A Geometric Mean Meningococcal Serum Bactericidal Antibody Titers

		Week 0 (Preprimary)		Week 4 (28 d Postprimary)		Week 40 (Prebooster)		Week 44 (28 d Postbooster)		Week 52 (1 y Postprimary)^a^		Week 104 (2 y Postprimary)^b^		Long-term Follow-up Study (5 y Postprimary)^c^	
Vaccine Group		SBA GMT													
Primary	Revaccination	No.	GMT (95% CI)	No.	GMT (95% CI)	No.	GMT (95% CI)	No.	GMT (95% CI)	No.	GMT (95% CI)	No.	GMT (95% CI)	No.	GMT (95% CI)
PsA-TT	PsA-TT	201	14.3 (9.9–20.7)	198	6234.5 (4947.9–7855.7)	63	1130.6 (666.0–1919.2)	58	10037.4 (7884.5–12778.2)	61	4485.8 (3579.6–5621.4)	61	2720.8 (1960.1–3776.8)	26	3781.2 (2410.4–5931.4)
	Hib-TT					65	801.3 (457.6–1403.1)	64	1649.1 (1022.7–2659.3)	65	1035.0 (678.4–1578.9)	64	1313.7 (875.1–1971.9)	29	2363.8 (1256.7–4446.1)
Hib-TT	PsA-TT	199	12.6 (8.7–18.2)	195	60.9 (39.8–93.2)	63	42.6 (20.3–89.4)	58	9342.9 (7043.8–12392.4)	58	3722.5 (2551.4–5431.3)	56	2527.6 (1796.9–3555.5)	26	3681.7 (2325.0–5830.0)
	Hib-TT					65	51.2 (23.5–111.5)	60	268.1 (128.0–561.5)	61	168.1 (81.0–349.1)	61	607.1 (338.3–1089.7)^d^	26	5949.1 (4242.4–8342.4)^d^
		SBA Titer ≥128													
		no./No.	% (95% CI)	no./No.	% (95% CI)	no./No.	% (95% CI)	no./No.	% (95% CI)	no./No.	% (95% CI)	no./No.	% (95% CI)	no./No.	% (95% CI)
PsA-TT	PsA-TT	70/201	34.8 (28.3–41.8)	193/198	97.5 (94.2–99.2)	58/63	92.1 (82.4–97.4)	58/58	100.0 (93.8–100.0)	61/61	100.0 (94.1–100.0)	60/61	98.4 (91.2–100.0)	26/26	100.0 (86.8–100.0)
	Hib-TT					58/65	89.2 (79.1–95.6)	61/64	95.3 (86.9–99.0)	62/65	95.4 (87.1–99.0)	62/64	96.9 (89.2–99.6)	28/29	96.6 (82.2–99.9)
Hib-TT	PsA-TT	61/199	30.7 (24.3–37.6)	112/195	57.4 (50.2–64.5)	33/63	52.4 (39.4–65.1)	58/58	100.0 (93.8–100.0)	57/58	98.3 (90.8–100.0)	55/56	98.2 (90.4–100.0)	26/26	100.0 (86.8–100.0)
	Hib-TT					34/65	52.3 (39.5–64.9)	46/60	76.7 (64.0–86.6)	44/61	72.1 (59.2–82.9)	53/61	86.9 (75.8–94.2)	26/26	100.0 (86.8–100.0)

Abbreviations: CI, confidence interval; GMT, geometric mean titer; Hib, *Haemophilus influenzae* type 1; PsA, polysaccharide; SBA, serum bactericidal antibody; TT, tetanus toxoid.

^a^ At 1-year postprimary comparison of SBA GMTs: Hib-TT/Hib-TT vs Hib-TT/PsA-TT, *P* < .0001; Hib-TT/Hib-TT vs PsA-TT/Hib-TT, *P* < .0001; Hib-TT/Hib-TT vs PsA-TT/PsA-TT, *P* < .0001; Hib-TT/PsA-TT vs PsA-TT/Hib-TT, *P* < .0001; PsA-TT/PsA-TT vs PsA-TT/Hib-TT, *P* < .0001.

^b^ At 2-year postprimary comparison of SBA GMTs: Hib-TT/Hib-TT vs Hib-TT/PsA-TT, *P* = .0001; Hib-TT/Hib-TT vs PsA-TT/Hib-TT, *P* = .0279; Hib-TT/Hib-TT vs PsA-TT/PsA-TT, *P* < .0001; PsA-TT/Hib-TT vs Hib-TT/PsA-TT, *P* = .0225 ; PsA-TT/Hib-TT vs PsA-TT/PsA-TT, *P* = .0042.

^c^ At 5-year postprimary comparison of SBA GMTs: Hib-TT/Hib-TT vs PsA-TT/Hib-TT, *P* = .0118.

^d^ From 2 to 5 years post–primary vaccination, SBA GMTs significantly increased for Hib-TT/Hib-TT (*P* = .0004).

The SBA GMTs of those vaccinated with Hib-TT followed by PsA-TT (Hib-TT/PsA-TT) were higher (*P* < .0001) than the GMTs of those who received PsA-TT followed by Hib-TT (PsA-TT/Hib-TT), with GMTs of 3722.5 (95% CI, 2551.4–5431.3) and 1035.0 (95% CI, 678.4–1578.9), respectively.

The SBA GMT at 1 year after primary vaccination in those who received 2 doses of PsA-TT (PsA-TT/PsA-TT) was statistically significantly higher than that in those who only received 1 dose of PsA-TT at primary vaccination (*P* < .0001) and similar to those who received 1 dose of PsA-TT at revaccination, with GMTs of 4485.8 (95% CI, 3579.6–5621.4), 3722.5 (2551.4–5431.3), and 1035.0 (95% CI, 678.4–1578.9) in the PsA-TT/PsA-TT, Hib-TT/PsA-TT, and PsA-TT/Hib-TT groups, respectively.

At 2 years after primary vaccination, the SBA GMT in those who had not received PsA-TT (Hib-TT/Hib-TT) was significantly lower than that of the 3 groups that had received PsA-TT (*P* = .0001 for Hib-TT/Hib-TT vs Hib-TT/PsA-TT; *P* = .0279 for Hib-TT/Hib-TT vs PsA-TT/Hib-TT; and *P* < .0001 for Hib-TT/Hib-TT vs PsA-TT/PsA-TT). The GMTs of the 2 groups that received a dose of PsA-TT at revaccination were similar at 2 years after primary vaccination, with GMTs of 2720.8 (95% CI, 1960.1–3776.8) and 2527.6 (95% CI, 1796.9–3555.5) in the PsA-TT/PsA-TT and Hib-TT/PsA-TT groups, respectively. However, the GMT of the group that received PsA-TT only at primary vaccination was lower (1313.7 [95% CI, 875.1–1971.9]; *P* = .0225 for PsA-TT/Hib-TT vs Hib-TT/PsA-TT and *P* = .0042 for PsA-TT/Hib-TT vs PsA-TT/PsA-TT). At 5 years after primary vaccination, the GMT of those who had not received PsA-TT in the initial study period but received PsA-TT at the end of the initial study (Hib-TT/Hib-TT) was statistically significantly higher than that in those vaccinated with only 1 dose of PsA-TT at primary vaccination (*P* = .0118). At the same time point, there was no significant difference in the GMTs of the Hib-TT/Hib-TT group and those who received PsA-TT at revaccination only (Hib-TT/PsA-TT) and those who received 2 doses of PsA-TT (PsA-TT/PsA-TT). From 2 to 5 years after primary vaccination, the SBA GMTs of the Hib-TT/Hib-TT group significantly increased (*P* = .004).

At all time points, a high percentage (>95%) of subjects in all groups that had received PsA-TT had SBA titers ≥128. In those who had not received PsA-TT (ie, received Hib-TT/Hib-TT), a lower percentage of subjects had SBA titers ≥128, with 72.1% (95% CI, 59.2%–82.9%) and 86.9% (95% CI, 75.8%–94.2%) of subjects achieving this level at 1 year and 2 years after primary vaccination, respectively; the percentage increased to 100% (95% CI, 86.8%–100.0%) at 5 years after primary vaccination (3 years after subjects in the Hib-TT/Hib-TT group received PsA-TT at the end of the initial study period).

### Group A–Specific IgG

The group A–specific IgG GMCs (µg/mL) and proportion of subjects with group A–specific IgG ≥2 µg/mL of each study group are shown in Table 2.

**Table 2.** Summary of Endpoints of Meningococcal Geometric Mean Group A–Specific IgG Concentrations

		Week 0 (Preprimary)		Week 4 (28 d Postprimary)		Week 40 (Prebooster)		Week 44 (28 d Postbooster)		Week 52 (1 y Postprimary)^a^		Week 104 (2 y Postprimary)^b^		Long-term Follow-up Study (5 y Postprimary)^c^	
Vaccine Group		Group A–Specific IgG GMCs													
Primary	Re-Vaccination	No.	GMC (95% CI)	No.	GMC (95% CI)	No.	GMC (95% CI)	No.	GMC (95% CI)	No.	GMC (95% CI)	No.	GMC (95% CI)	No.	GMC (95% CI)
PsA-TT	PsA-TT	200	0.1 (.1–.1)	199	18.2 (16.0–20.7)	63	1.0 (.8–1.4)	58	38.1 (25.5–57.2)	61	7.3 (5.4–9.8)	61	3.7^d^ (2.7–5.0)	26	2.0^d^ (1.4–2.8)
	Hib-TT					65	1.0 (.7–1.4)	64	1.1 (.7–1.6)	65	0.9 (.6–1.3)	64	0.9 (.7–1.3)	29	1.1 (.7–1.6)
Hib-TT	PsA-TT	198	0.1 (.1–.2)	196	0.1 (.1–.1)	63	0.1 (.1–.2)	58	15.4 (11.7–20.2)	58	1.6 (1.2–2.1)	56	1.2 (.9–1.7)	26	1.2 (.9–1.6)
	Hib-TT					65	0.2 (.1–.2)	60	0.2 (.1–.2)	61	0.2 (.1–.3)	61	0.3^d^ (.2–.5)	26	2.1^d^ (1.4–3.3)
		Group A–Specific IgG Concentration ≥2 µg/mL													
		no./No.	% (95% CI)	no./No.	% (95% CI)	no./No.	% (95% CI)	no./No.	% (95% CI)	no./No.	% (95% CI)	no./No.	% (95% CI)	no./No.	% (95% CI)
PsA-TT	PsA-TT	3/200	1.5 (.3–4.3)	198/199	99.5 (97.2–100.0)	16/63	25.4 (15.3–37.9)	55/58	94.8 (85.6–98.9)	55/61	90.2 (79.8–96.3)	44/61	72.1 (59.2–82.9)	14/26	53.8 (33.4–73.4)
	Hib-TT			24/65	36.9 (25.3–49.8)	23/64	35.9 (24.3–48.9)	16/65	24.6 (14.8–36.9)	19/64	29.7 (18.9–42.4)	7/29	24.1 (10.3–43.5)		
Hib-TT	PsA-TT	2/198	1.0 (.1–3.6)	3/196	1.5 (.3–4.4)	0/63	0.0 (.0–5.7)	56/58	96.6 (88.1–99.6)	24/58	41.4 (28.6–55.1)	15/56	26.8 (15.8–40.3)	7/26	26.9 (11.6–47.8)
	Hib-TT					2/65	3.1 (.4–10.7)	2/60	3.3 (.4–11.5)	2/61	3.3 (.4–11.3)	7/61	11.5 (4.7–22.2)	14/26	53.8 (33.4–73.4)

Abbreviations: CI, confidence interval; GMC, geometric mean concentration; Hib, *Haemophilus influenzae* type 1; IgG, immunoglobulin G; PsA, polysaccharide; SBA, serum bactericidal antibody; TT, tetanus toxoid.

^a^ At 1-year postprimary comparison of IgG GMCs: Hib-TT/Hib-TT vs Hib-TT/PsA-TT, *P* < .0001; Hib-TT/Hib-TT vs PsA-TT/Hib-TT, *P* < .0001; Hib-TT/Hib-TT vs PsA-TT/PsA-TT, *P* < .0001; Hib-TT/PsA-TT vs PsA-TT/Hib-TT, *P* = .0037; PsA-TT/PsA-TT vs Hib-TT/PsA-TT, *P* < .0001; PsA-TT/PsA-TT vs PsA-TT/Hib-TT, *P* < .0001.

^b^ At 2-year postprimary comparison of IgG GMCs: Hib-TT/Hib-TT vs Hib-TT/PsA-TT, *P* < .0001; Hib-TT/Hib-TT vs PsA-TT/Hib-TT, *P* < .0001; Hib-TT/Hib-TT vs PsA-TT/PsA-TT, *P* < .0001; PsA-TT/PsA-TT vs Hib-TT/PsA-TT, *P* < .0001; PsA-TT/PsA-TT vs PsA-TT/Hib-TT, *P* < .0001.

^c^ At 5-year postprimary comparison of IgG GMCs: PsA-TT/PsA-TT vs PsA-TT/Hib-TT, *P* = .0110 ; Hib-TT/Hib-TT vs PsA-TT/Hib-TT, *P* = .0089 ; PsA-TT/PsA-TT vs Hib-TT/PsA-TT, *P* = .0483; Hib-TT/Hib-TT vs Hib-TT/PsA-TT, *P* = .0386.

^d^ From 2 to 5 years post–primary vaccination, IgG GMCs significantly increased for PsA-TT/PsA-TT (*P* = .0149) and Hib-TT/Hib-TT (*P* < .0001).

At 1 year and 2 years after primary vaccination, the group A–specific IgG GMC in all 3 groups that received PsA-TT was significantly higher than that of those who had not received PsA-TT (Hib-TT/Hib-TT) (*P* < .0001 for all 3 pairwise comparisons at 1 year and 2 years after primary vaccination).

The group A–specific IgG GMC of the Hib-TT/PsA-TT group was significantly higher than that of the PsA-TT/Hib-TT group at 1 year after primary vaccination, with GMCs of 1.6 (95% CI, 1.2–2.1) and 0.9 (95% CI, .6–1.3) (*P* = .0037).

This effect was not found at 2 years after vaccination, though, with GMCs of 1.2 (95% CI, .9–1.7) and 0.9 (95% CI, .7–1.3) in the Hib-TT/PsA-TT and PsA-TT/Hib-TT groups, respectively. At 1 year postvaccination, the group that received 2 doses of PsA-TT had higher group A–specific IgG GMCs (7.3 [95% CI, 5.4–9.8]) than the Hib-TT/PsA-TT group (1.6 [95% CI, 1.2–2.1]) (*P* < .0001). The GMC of the 2-dose PsA-TT group was also higher than that of Hib-TT/PsA-TT at 2 years following vaccination (*P* < .0001), with GMCs of 3.7 (95% CI, 2.7–5.0) and 1.2 (95% CI, .9–1.7), respectively.

At 5 years after primary vaccination, the GMCs of the PsA-TT/PsA-TT and Hib-TT/Hib-TT (who had received PsA-TT at the end of the initial study period) groups were significantly higher than the GMCs in the PsA-TT/Hib-TT and Hib-TT/PsA-TT groups (*P* = .0110 for PsA-TT/PsA-TT vs PsA-TT/Hib-TT; *P* = .0089 for Hib-TT/Hib-TT vs PsA-TT/Hib-TT; *P* = .0483 for PsA-TT/PsA-TT vs Hib-TT/PsA-TT; and *P* = .0386 for Hib-TT/Hib-TT vs Hib-TT/PsA-TT), with significant increases in the PsA-TT/PsA-TT (*P* = .0149) and Hib-TT/Hib-TT (*P* < .0001) groups from 2 years following primary vaccination to 5 years following primary vaccination.

## DISCUSSION

Children vaccinated with PsA-TT demonstrated sustained SBA levels from 1 year to 5 years following vaccination that were significantly above baseline levels, with the majority of subjects having SBA titers ≥128.

Direct protection from meningococcal disease and vaccine effectiveness against susceptibility to disease have been shown to be associated with high vaccine coverage inducing herd protection in the population and persistence of circulating antibodies rather than with evidence of immunologic priming (induction of memory with high booster responses) [13]. Earlier expectations that the ability to prime for immunologic memory could be a key determinant in direct protection to disease [14, 15] have been revisited in light of the extensive experience with widespread use of meningococcal C conjugate vaccines and that of conjugate vaccines against other encapsulated bacteria such as Hib [15–17]. The ability to generate a booster response on direct exposure was found not to be sufficient to ensure protection from disease, as antibody levels in subjects with vaccine failure showed evidence of an anamnestic immune response [16, 17]. However, the presence of circulating bactericidal antibodies in adequate quantities, along with interruption of carriage, appears to be critical for the protection against invasive disease caused by *Neisseria meningitidis.* The assessment of their persistence over time has now become an essential component of the evaluation of new meningococcal vaccines to better inform decisions on schedules and vaccination programs.

Sow et al [9] reported a decline in antibody levels in the 10 months following PsA-TT vaccination, but the levels reported were higher than those vaccinated with a PsACWY vaccine. The results reported here show that there was no further antibody decline in those vaccinated with 1 dose of PsA-TT when aged 12–23 months, with similar SBA GMTs and IgG GMCs measured at 1, 2, and 5 years following vaccination, both of which were significantly higher than those measured at baseline. The group A meningococcal SBA GMTs measured in this trial at 1 year following 1 dose of PsA-TT were comparable to those measured in Finnish children vaccinated at a similar age with a quadrivalent meningococcal ACWY conjugate vaccine (ACWY-TT, Nimenrix, GlaxoSmithKline) in which an SBA GMT of 967 (95% CI, 843–1109.3) at 1 year postvaccination was observed [18]. However, at the later persistence time points of 2 and 3 years following vaccination, a decline in GMTs was observed in the trial of Vesikari et al [18], whereas similar GMTs were measured at 1, 2, and 5 years postvaccination in this trial. Despite the decline in GMTs, the majority of subjects in the trial of Vesikari et al had SBA titers ≥128 at all persistence time points; this was also true of the results reported here. It is of note that the baseline immunogenicity characteristics of the Finnish child population [18] were very different to those of our trial reported here and may contribute to the differences observed.

Difference in the SBA GMTs was observed at 1 and 2 years postvaccination between those who received 1 dose of PsA-TT at primary vaccination and those who received 2 doses. However, the same difference was not observed when a single dose of PsA-TT was administered at an older age of 22–33 months, at the revaccination visit. Differences between the groups that received 1 and 2 doses were also evident with the IgG GMCs, with those who received 2 doses having higher IgG GMCs at 2 and 5 years after primary vaccination. This is in contrast to the results observed by Klein et al [19], which showed no difference in the group A meningococcal SBA GMTs 1 year following vaccination with 1 dose of quadrivalent meningococcal ACWY vaccine at 12 months of age or 2 doses at 9 and 12 months of age, with group A meningococcal SBA GMTs of 259.7 (95% CI, 191.4–352.3) and 237.2 (95% CI, 187.0–301.0) in those who received 1 or 2 doses, respectively. The SBA GMTs and IgG GMCs of the Hib-TT/Hib-TT group that received PsA-TT at the end of the initial trial period at an older age of 3–4 years were similar to those of the group that received 2 doses of PsA-TT. Despite observed differences in the persisting antibody levels in those who received 1 or 2 doses of PsA-TT, both the SBA GMTs and IgG GMCs at 5 years after primary vaccination were significantly above baseline for all groups in which subjects received PsA-TT during the initial study or at the end of the initial study.

PsA-TT has been introduced into several countries of the African meningitis belt via mass vaccination campaigns in which individuals aged 1–29 years received 1 dose. PsA-TT has been shown to have had a major impact on MenA disease and carriage in Chad [20]. This study and others [21] have demonstrated the persistence of antibodies following PsA-TT; however, ongoing active surveillance is needed to establish the longer-term duration of protection provided by PsA-TT. In conclusion, we have demonstrated that 5 years following a single dose of PsA-TT in African children aged 12–23 months, the majority are still protected.
